# Adrenomedullin Inhibits the Efficacy of Combined Immunotherapy and Targeted Therapy in Biliary Tract Cancer by Disrupting Endothelial Cell Functions

**DOI:** 10.1111/jcmm.70460

**Published:** 2025-03-12

**Authors:** Zhengfeng Xuan, Haoran Hu, Jian Xu, Xiaowei Ling, Long Zhang, Wenzhu Li, Junda Li, Chan Zhu, Yunjie Song, Xing Zhang, Jianhua Rao, Yong Wang, Feng Cheng

**Affiliations:** ^1^ Hepatobiliary Center, the First Affiliated Hospital of Nanjing Medical University & Research Unit of Liver Transplantation and Transplant Immunology Chinese Academy of Medical Sciences Nanjing Jiangsu China; ^2^ Department of General Surgery Changzhou Jintan District People's Hospital Changzhou Jiangsu China; ^3^ Jiangsu Simcere Diagnostics Co. Ltd., Nanjing Simcere Medical Laboratory Science Co. Ltd. The State Key Laboratory of Neurology and Oncology Drug Development Nanjing China

**Keywords:** adrenomedullin, antivascular therapy, biliary tract cancer, calcitonin receptor‐like receptor, immunotherapy, vascular endothelial growth factor

## Abstract

The global incidence of biliary tract cancer (BTC) is on the rise, presenting a substantial healthcare challenge. The integration of immune checkpoint inhibitors (ICIs) with molecularly targeted therapies is emerging as a strategy to enhance immune responses. However, the efficacy and underlying mechanisms of these treatments in BTC are still largely unexplored. In this study, tissue samples from 19 BTC patients treated with camrelizumab and apatinib were analysed using the NanoString 289‐panel to identify key molecular biomarkers. Comparative analyses and subsequent experimental validations, including cell‐based assays and histopathological examinations, identified adrenomedullin (ADM) as a critical molecular marker associated with treatment efficacy and poor prognosis. ADM has been shown to promote BTC cell proliferation, migration and angiogenesis, primarily by interacting with vascular endothelial growth factor (VEGF) and increasing AKT phosphorylation. Furthermore, ADM disrupts endothelial cell barrier function via the calcitonin receptor‐like receptor (CRLR) and vascular endothelial (VE)‐cadherin signalling pathway. Preclinical inhibition of ADM or CRLR resulted in suppressed tumour growth. Additionally, elevated ADM expression was correlated with increased tumour‐infiltrating immune cells and higher immune checkpoint expression. These findings suggest that ADM plays a pivotal role in resistance to immunotherapy and anti‐angiogenic treatment in BTC, and thus, targeting ADM may offer a promising therapeutic approach to enhance treatment efficacy.

## 1 Introduction

Biliary tract cancer (BTC) constitutes about 3% of all digestive malignancies, encompassing intrahepatic cholangiocarcinoma (ICC), gallbladder cancer (GBC) and cholangiocarcinoma (CHOL) [1]. Diagnosis typically occurs at middle to advanced stages, presenting complex conditions and poor prognoses, with a current 5‐year survival rate of 5%–15% [2]. At present, chemotherapy has been the mainstay treatment for advanced unresectable BTC, yet its efficacy is limited and safety concerns impact patient survival and quality of life [3]. While advances in transarterial chemoembolisation, drug therapy and radiotherapy have been notable, monotherapy has reached a ‘ceiling effect’, limiting further substantial improvements in outcomes, demonstrating a need for improving treatment strategies.

Significant advancements in immune checkpoint blockade (ICB) therapy, particularly with anti‐PD‐1/L1 and anti‐CTLA4 antibodies, have markedly extended survival across various cancers, establishing immunotherapy as a cornerstone in oncology [4, 5]. However, a substantial portion of cancer patients fail to benefit from these treatments. Vascular abnormalities are a characteristic feature in most solid tumours, promoting immune evasion through heightened levels of proangiogenic factors such as vascular endothelial growth factor (VEGF) and angiopoietin 2 [6]. Recently, combination therapies involving immunotherapy and anti‐angiogenic agents have shown promise in treating solid tumours [7, 8]. Strategic targeting of angiogenesis can enhance therapeutic efficacy by normalising aberrant tumour vasculature, facilitating increased infiltration of immune effector cells into tumours and converting the inherently immunosuppressive tumour microenvironment (TME) into an immunosupportive milieu [6, 9]. Immunotherapy hinges on the accumulation and activation of immune effector cells within the TME, with evidence suggesting reciprocal regulation between immune responses and vascular normalisation [10]. Yet, the specific efficacy of immune checkpoint inhibitors and anti‐angiogenic therapies in BTC patients remains uncertain [6]. While angiogenesis inhibitors like Apatinib can diminish tumour blood supply, tumour cells may adapt by accessing nutrients through alternative pathways, potentially limiting therapeutic efficacy or fostering resistance [11]. Enhancing the effectiveness of immune checkpoint inhibitors combined with anti‐angiogenic therapies represents a promising avenue for BTC treatment. Achieving this goal necessitates identifying biomarkers that can predict treatment response, underscoring the need for a comprehensive understanding of the tumour immune microenvironment (TIME) unique to BTC.

Adrenomedullin (ADM) is a powerful vasodilatory peptide commonly present in various tumours [12]. Although ADM was initially isolated from human adrenal tumours, recent studies have demonstrated that ADM mRNA is overexpressed in multiple tumour tissues and promoting angiogenesis and tumour growth, and its expression is closely associated with the histological grading of various tumours via regulating the production of vascular endothelial growth factor (VEGF) [12, 13]. Additionally, research indicates that ADM can disrupt intercellular adhesion of endothelial cells, promoting unstable angiogenesis and hindering the delivery of anti‐tumour drugs [14]. However, it remains unclear whether ADM can influence the efficacy of immunotherapy and anti‐angiogenic treatment in BTC.

Herein, we utilised NanoString 289‐panel analysis to discern genetic characteristic disparities between BTC patients who responded versus those who did not combine immunotherapy and anti‐angiogenic treatments. Our investigation identified ADM as a pivotal biomarker that restricts immune cell infiltration into the tumour core, thereby fostering drug resistance. In vitro studies demonstrated that blocking ADM attenuates biological functions of BTC cells, while in vivo experiments showed reduced tumour growth upon ADM blockade. Moreover, our findings elucidate the interaction between tumour cells and endothelial cells mediated by the ADM/CRLR signalling pathway. These results collectively underscore the influence of ADM within the TME on the efficacy of immunotherapy and anti‐angiogenic treatments. These results suggest that targeting ADM or disrupting the tumour‐endothelial cell interaction, in combination with immunotherapy and anti‐angiogenic approaches, could significantly enhance therapeutic outcomes.

## 2 Methods

### 2.1 Clinical Specimens and Immunohistochemistry (IHC)

The tissues were collected from 19 patients diagnosed with ICC and GBC who underwent surgical treatment at the First Affiliated Hospital of Nanjing Medical University from 2018 to 2021 (2023‐SR‐130). The patients were followed up until death or 28 December 2022. Each tissue sample underwent dewaxing and rehydration, followed by preparation using an ethanol gradient. Antigen retrieval was performed by heating the samples at 95°C for 20 min in citrate buffer (10 mM, pH 6.0). The samples were then incubated overnight at 4°C with a polyclonal antibody against rabbit anti‐ADM pAb (1:50, 10778‐1‐AP, Proteintech). Afterward, the samples were incubated with a secondary antibody for 1 h. IHC results were evaluated by *H*‐score, a semiquantitative approach. Staining intensity was classified as negative (−), weak (+), moderate (++) or strong (+++). The *H*‐score was calculated using the formula: 1 × (% weak staining cells) + 2 × (% moderate staining cells) + 3 × (% strong staining cells). Written informed consent was obtained from all participants. Our study received approval from the Ethics Committee of the First Affiliated Hospital of Nanjing Medical University. Figure S1, along with Tables S1 and S2, presents the patient selection flowchart and detailed demographic information of the study cohort.

### 2.2 NanoString 289‐Panel RNA Sequencing

Total RNA was extracted from a 5‐μm thick FFPE slice sample, and 100 ng RNA was hybridised to a beta version of the NanoString PanCancer code set, which was read on the nCounter platform (NanoString Technologies, Seattle, WA). The expressions of 289 cancer immune response‐related genes were assessed (Table S3). For each sample, quality control (QC) indicators included the imaging QC, binding density QC, positive control linearity QC and positive control limit of detection QC. The raw data of each sample and gene were standardised against internal controls to eliminate technical variability in the assay, and then counts were normalised to the geometric mean of endogenous housekeeping genes followed by log_2_ transformation.

### 2.3 Reagents and Cell Culture

Human intrahepatic cholangiocarcinoma cell line (HuCCT1) and gallbladder cancer cell line (NOZ) were obtained from Health Sciences Research Bank, Osaka, Japan. The following cells were obtained from the Cell Bank of the Chinese Academy of Sciences (Shanghai, China): HIBEC, RBE, HGEpc and GBC‐SD. Culture temperature was 37°C, 5% CO_2_ and the medium contained 10% fetal bovine serum (Gibco) and 1% penicillin G/streptomycin. The GBC‐SD cells were cultured in RPMI 1640 medium (Gibco), and the HIBEC, GBC‐SD cells were cultured in RPMI 1640 medium (Gibco); HIBEC, HuCCT1, RBE, HGEpc and NOZ cells were cultured in DMEM medium (Gibco).

### 2.4 Cell Transfection

ADM was silenced with small interfering RNAs (siRNAs) with Lipofectamine 3000 (Thermo Fisher), following the manufacturer's protocol. The target sequences were siADM (target sequence, GGCCUAGCAAUCGCU UUAATT); shCRLR (target sequence, CCTTCCATTTCTACTGTATAA). ADM shRNA was obtained from GenePharma (Shanghai, China). The shRNA targeting ADM was synthesised according to the sequences of siADM and inserted into the GV493 vector. Empty vectors were used as control. HuCCT1 and NOZ cells were infected by concentrated lentivirus at a multiplicity of infection (MOI) of 20 for 72 h, using HiTransG A as recommended by the manufacturer's protocol. Stable cell lines were generated by puromycin (1 μg/mL) treatment for 1 week. Transfection efficiency was verified by qRT‐PCR and western blotting.

### 2.5 Quantitative Real Time PCR (qRT‐PCR)

Total RNA was isolated from GBC/ICC tissues and cell lines using Trizol RNA isolation reagent (Invitrogen) according to the manufacturer's instructions and quantified by Nanodrop One (Thermo Fisher Scientific, Madison, USA), followed by reverse transcription into cDNA using HiScript II Q RT SuperMix for qPCR (Vazyme, Jiangsu, China). qRT‐PCR was carried out with cDNA in triplicate using ChamQ SYBR qPCR Master Mix (Vazyme, China) on the QuantStudio 5 Real‐Time PCR System (Thermo Fisher Scientific). Gene expression values were normalised against that of Beta‐Actin. Data were analysed with the 2^ΔΔ*C*^_t_ method. The primers were as follows: ADM (F: 5′‐ATGAAGCTGGTTTCCGTCG‐3′; R: 5′‐GACATCCGCAGTTCCCTCTT‐3′); β‐actin (F: 5′‐CGTCACCAACTGGGACGA‐3′; R: 5′‐ATGGGGGAGGGCATACC‐3′); VEGF (F: 5′‐GAGGGCAGAATCATCACGAAG‐3′; R: 5′‐TGTGCTGTAGGAAGCTCATCTCTC‐3′); PCNA (F: 5′‐TTGCACGTATATGCCGAGACC‐3′; R: 5′‐GGTGAACAGGCTCATTCATCTCT‐3′); F11r (F: 5′‐TCTCTTCACGTCTATGATCCTGG‐3′; R: 5′‐TTTGATGGACTCGTTCTCGGG‐3′); Cdh5 (F: 5′‐CCACTGCTTTGGGAGCCTT‐3′; R: 5′‐GGCAGGTAGCATGTTGGGG‐3′)； Ctnnd1 (F: 5′‐GTGGAAACCTACACCGAGGAG‐3′; R: 5′‐CGTCTAGTGGTCCCATCATCTG‐3′)； Gja1 (F: 5′‐ACAAGGTCCAAGCCTACTCCA‐3′; R: 5′‐CCGGGTTGTTGAGTGTTACAG‐3′)； Gjc1 (F: 5′‐AGATCCACAACCATTCGACATTT‐3′; R: 5′‐TCCCAGGTACATCACAGAGGG‐3′).

### 2.6 Protein Extraction and Western Blot (WB) Analysis

Whole protein extraction kit was purchased from Key GEN Biotech Co. Ltd. (Nanjing, China). For normal western blot, total protein was extracted from GBC cells using lysis buffer supplemented with 1% PMSF and proteinase inhibitor cocktail. Bicinchoninic acid (BCA) assay was used to measure the protein concentration. Equal amounts of protein were loaded on a 10% sodium salt‐polyacrylamide gel electrophoresis (SDS‐PAGE) and transferred to PVDF membranes (Millipore, Bedford, MA). Then, the blots were blocked in 5% skimmed milk with 0.1% Tween 20 for 1 h at room temperature followed by incubation at 4°C overnight with primary antibodies. The membranes were washed with tris‐buffered saline and tween and then incubated with secondary antibody at room temperature for 2 h. The blots were detected by ECL chemiluminescence kit (NCM Biotech). The western blot analysis involved the use of the following antibodies at specific dilutions: rabbit anti‐ADM pAb (10778‐1‐AP, Proteintech), rabbit anti‐VEGF pAb (19003‐1‐AP, Proteintech), rabbit anti‐PCNA pAb (10205‐2‐AP, Proteintech), rabbit anti‐Akt mAb (4691 T, Cell Signalling Technology), rabbit anti‐phospho‐Akt mAb (4060 T, Cell Signalling Technology), rabbit anti‐VE‐cadherin pAb (ab33168, Abcam), rabbit anti‐VE‐Cadherin (phospho Y685) pAb (ab119785, Abcam), rabbit anti‐Src Family pAb (2108S, Cell Signalling Technology), rabbit Anti‐SRC Family (phospho Y418) mAb (ab40660, Abcam) and rabbit anti‐β‐actin mAb (AC026, ABclonal).

### 2.7 CCK‐8 Assay and Colony Formation

Cell proliferation was assessed using CCK‐8 assays. Approximately 1000 cells per well were seeded into 96‐well plates. Next, 10 μL of the CCK‐8 reagent (Beyotime Biotechnology) was added to each well, and the microplate was incubated for 24 h at 37°C. The OD450 was measured using a microplate reader (Multiskan Sky, Thermofisher) every 24 h for 96 h. Stably transfected HuCCT1 and NOZ cells were seeded at a density of 1000 cells per well in six‐well plates and cultured in DMEM supplemented with 10% fetal bovine serum (FBS). After 2 weeks of incubation, the plates were harvested. Colonies were fixed with 4% paraformaldehyde (Sevicebio, Wuhan, China) for 25 min, followed by staining with 0.1% crystal violet (Beyotime). After washing with phosphate‐buffered saline (PBS), colony formation and proliferation were assessed through imaging and colony counting.

### 2.8 Wound Healing and Transwell Migration Assay

To perform the wound healing assay, cells were seeded into 6‐well plates and allowed to grow to a more than 90% confluency. The monolayer was then uniformly wounded using a 200‐μL pipette tip. After washing with fresh medium, the cells were incubated in a serum‐free medium for 48 h, and images were taken from 5 random fields of view using a microscope. The wound closure percentage was evaluated by comparing the changes in the wound area before and after 48 h. For HuCCT1/NOZ/HUVECs Transwell migration assay, 24‐well plates with 8 μm chamber inserts (Corning Life Science) were used. A total of 3 × 10^4^ cells were seeded in the upper chamber with a serum‐free medium in triplicate. The HUVECs were co‐incubated with PBS (as control), ADM (100 nM), ADM22‐52 (1 nM) or Apatinib (2 μM). Medium containing 10% FBS was added to the lower chamber as a chemo‐attractant. After incubation for 24 h, the cells in the chamber were removed by cotton swabs, and the cells below the membrane were fixed with 4% PFA, stained with 0.1% crystal violet for 10 min, and counted.

### 2.9 In Vitro Tube Formation Assays

The effect of ADM on angiogenesis in vitro was examined by tube formation assay. The wells of a 96‐well plate were coated with 50 μL of ice‐cold Growth Factor Reduced Matrigel (BD Bioscience, San Jose, CA) at 37°C for 1 h. HUVECs were seeded at a density of 5 × 10^4^ cells per well in 200 μL complete culture medium containing PBS (equal volume as control) ADM (100 nM) or ADM22‐52 (1 nM) or Apatinib (2 μM). After incubation for 24 h at 37°C with 5% CO_2_, the cultures were photographed, and the tube‐like structures were evaluated.

### 2.10 Animal Experiment

Four‐week‐old male BALB/c nude mice were obtained from GemPharmatech Co. Ltd. All animal experiments were approved by the Nanjing Medical University Ethics Committee (No. 2302013). The mice were housed under specific pathogen‐free (SPF) conditions, with a 12‐h light/12‐h dark cycle (150–300 lx), ambient temperature ranging from 20°C to 26°C and humidity maintained between 40% and 70%. The room was ventilated four times per hour. The mice were randomly divided into four groups, with five mice in each group. In the xenograft tumour growth experiment, sh‐ADM HuCCT1 cells, sh‐ADM NOZ cells, and their respective control cells (5 × 10^6^ cells in 100 μL PBS) were inoculated into the axillae of the mice. After 4 weeks, cervical dislocation was used for euthanasia. Tumour size was measured weekly using digital calipers. Tumour volume was calculated using the formula (width^2^ × length)/2, and tumour weight was recorded. Furthermore, euthanasia was performed under specific conditions, such as anticipated death, severe physical deterioration and ulceration or abnormal behaviour due to tumour burden.

### 2.11 Statistical Analysis

Statistical analyses were performed using SPSS 19.0 and GraphPad Prism 9.0 software. Data are presented as mean ± SD. The *t*‐test was used to analyse the differences between the two sample groups. Multiple group comparisons were performed by one‐way analysis of variance followed by Bonferroni's post hoc test. The expression of ADM in relation to the clinicopathological data of BTC patients was analysed using Pearson's *χ* ^2^ test. Survival probabilities were assessed using the Kaplan–Meier method and compared with the log‐rank test. Univariate and multivariate analyses were conducted using the Cox proportional hazards regression model. *p* value < 0.05 was considered statistically significant.

## 3 Results

### 3.1 ADM Is Associated With Immunotherapy and Anti‐Angiogenic Treatment Efficacy

**FIGURE 1:**
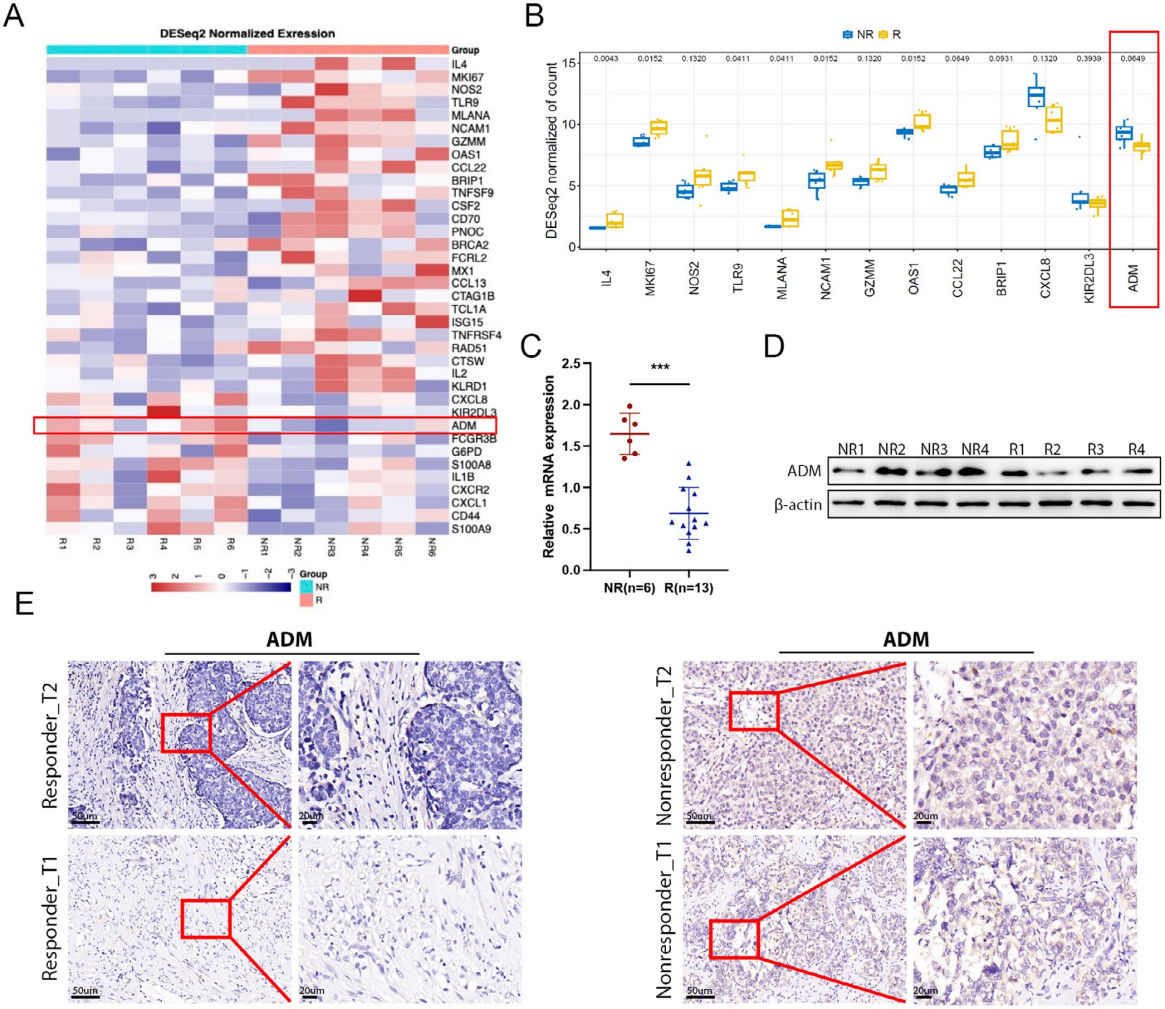
Identification of ADM and its upregulation in non‐responders. (A) The volcano plot illustrates the differentially expressed tumour markers. (B) Box plots depict the distribution of gene expression levels in tissues from responding and non‐responding patients. (C) Relative mRNA levels of ADM in tumours from 6 non‐responders and 13 responders as determined by qRT‐PCR. (D) ADM protein expression in tumour tissue from a representative non‐responding patient (NR) and a responding patient (R). (E) Representative images of ADM expression in tumour tissues from responding and non‐responding patients, detected using IHC staining. *p < 0.05, **p < 0.01, ***p < 0.001.

To assess the efficacy of immunotherapy and anti‐angiogenic treatment in BTC patients, we enrolled 19 individuals who received camrelizumab in combination with apatinib (Table S1). Subsequently, tissue samples were collected and analysed to compare transcriptional profiles between responders (R, *n* = 13) and non‐responders (NR, *n* = 6) (Figure 1A,B). Our investigation revealed abnormal upregulation of ADM expression in the non‐responder group compared to responders. We further validated these findings by quantifying ADM expression using qRT‐PCR in tissue samples from both groups (Figure 1C). Additionally, WB and IHC confirmed significantly higher levels of ADM protein in BTC tissues from non‐responders compared to responders (Figure 1D,E). These results suggested that ADM is related to immunotherapy and anti‐angiogenic treatment efficacy.

### 3.2 The Expression of ADM Correlates With Human Biliary Tract Cancer Progression

**FIGURE 2:**
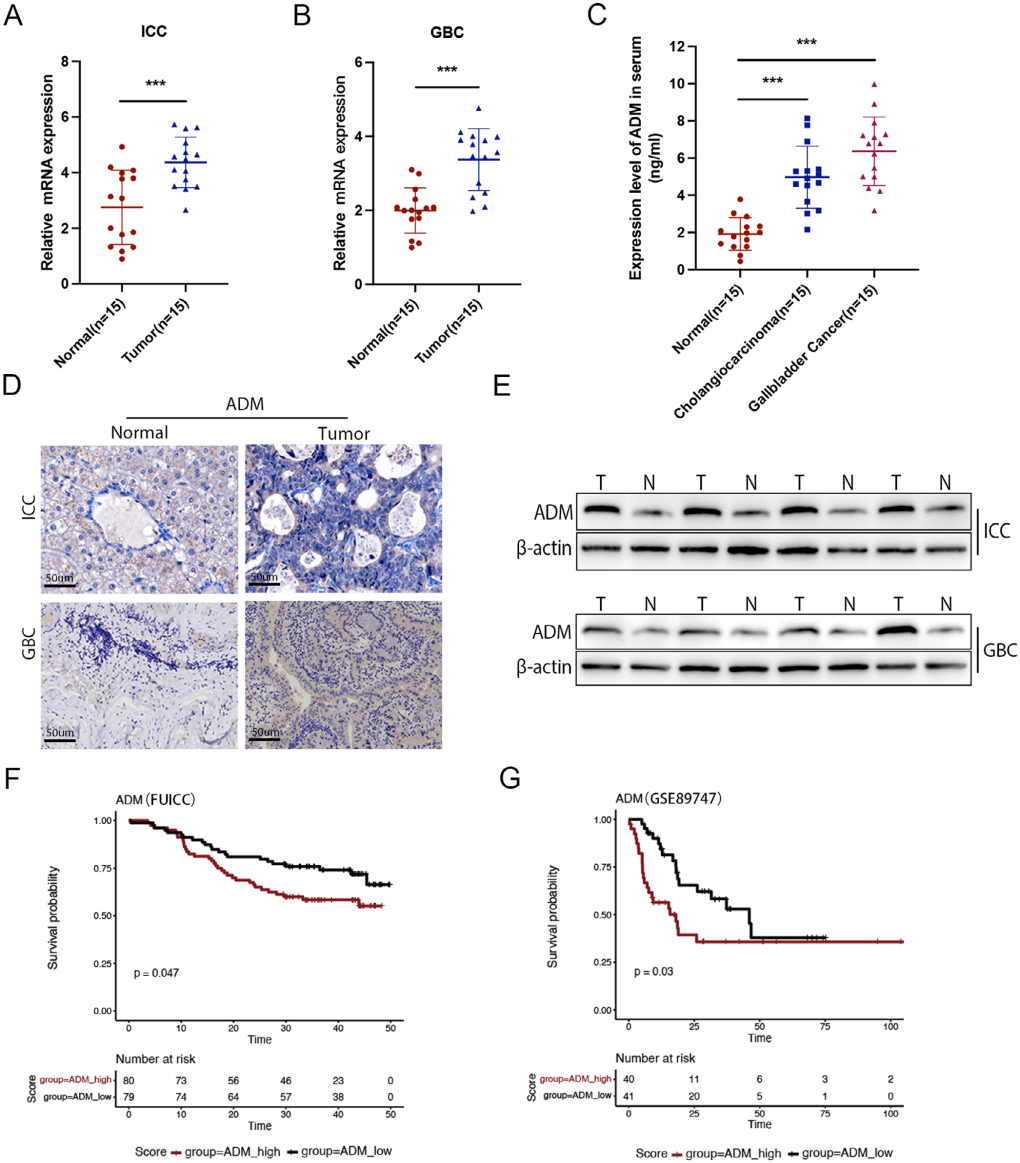
Clinical significance of ADM in human biliary tract cancer. (A, B) Relative mRNA levels of ADM in 15 paired intrahepatic ICC/GBC and normal tissues were measured by qRT‐PCR. (C) ADM levels in plasma from normal individuals and cancer patients were quantified by ELISA; *p*‐values were calculated using Student's *t*‐test. (D) Representative images of ADM expression in BTC tissues and adjacent non‐tumour tissues were obtained using IHC staining. (E) Protein expression of ADM in representative primary BTC tissues (T) and their paired non‐tumour tissues (N) is shown. (F, G) The Kaplan–Meier overall survival curve for ICC patients in the FU and GSE 89747 cohorts. Patients were stratified stratified by the median expression of ADM. *p < 0.05, **p < 0.01, ***p < 0.001.

To detect the potential role of ADM in BTC, we analysed the expression levels of ADM in human BTC tissues and adjacent non‐tumour tissues through performing qRT‐PCR experiments. The results showed that the expression level of ADM in tumour tissues was upregulated notably compared with adjacent non‐tumour tissues (Figure 2A,B). Although sample size is typically not a concern in basic experimental studies, a power calculation was performed on the key samples from both groups in the preliminary analysis. The calculated power values were 0.9484 and 0.9623, respectively, confirming that the study has sufficient power to detect a true effect. Consistently, the level of ADM in serum in the tissue samples from BTC patients was significantly higher than that in normal individuals (Figure 2C). Additionally, IHC staining was used to examine ADM expression in 30 pairs of BTC patients and controls. The results indicated that ADM staining was stronger in tumours compared to normal tissues (Figure 2D). Furthermore, protein levels of ADM were determined by protein blotting in 8 pairs of human BTC specimens and their matched non‐tumour tissues. ADM protein expression was found to be significantly higher in BTC tissues than in benign tissues (Figure 2E). The prognostic value of the ADM risk signature in ICC was further evaluated using Kaplan–Meier (KM) analysis. The results indicated that patients with a high ADM expression had significantly poorer overall survival (OS) (Figure 2F,G). These results indicated a potential role of ADM in the development of BTC.

### 3.3 ADM Promotes Biliary Tract Cancer Progression Both In Vitro and In Vivo

**FIGURE 3:**
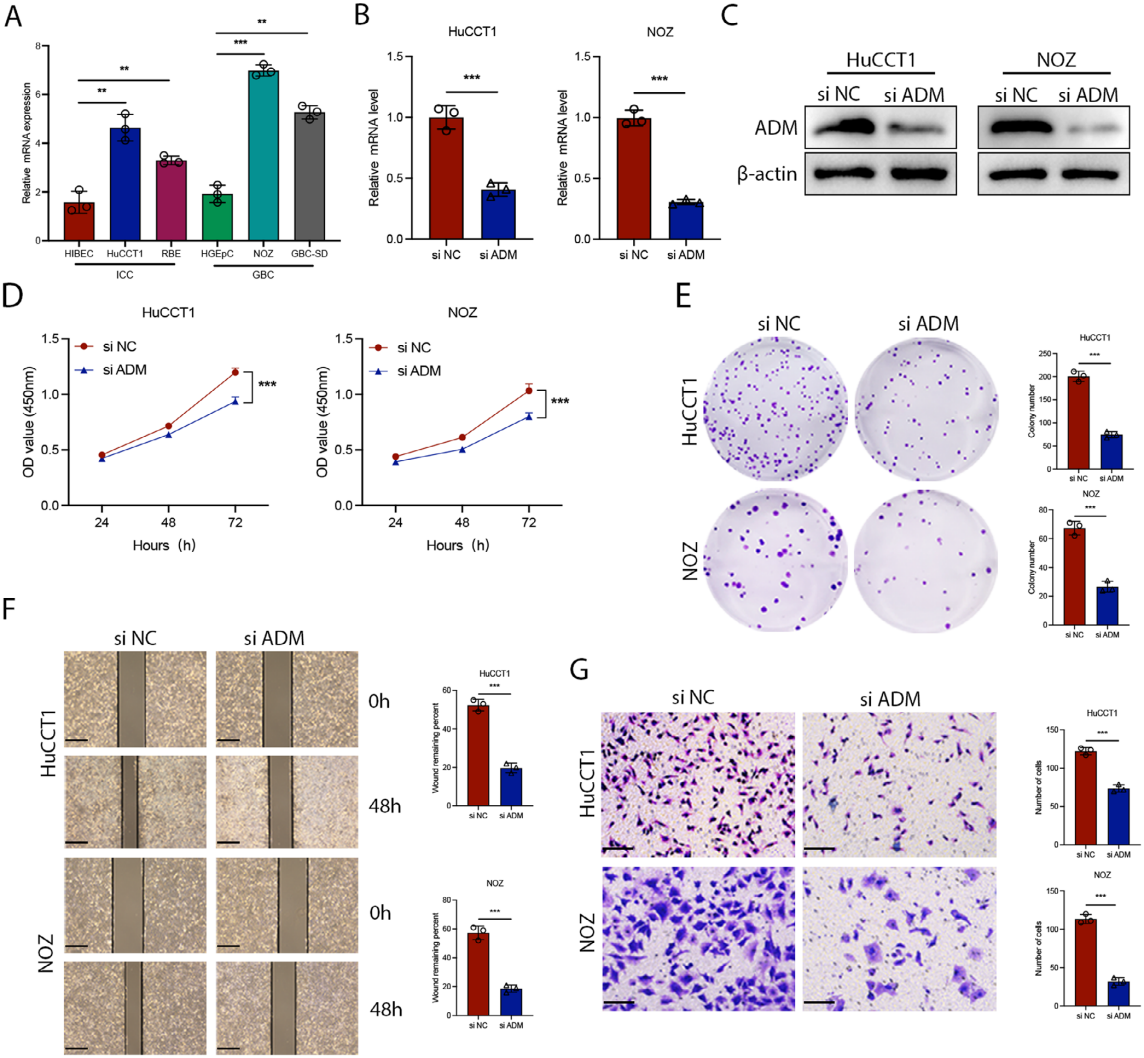
ADM promotes the proliferation and migration of BTC in vitro. (A) Relative mRNA levels of ADM in HIBEC, HuCCT1, RBE, HGEpC, NOZ and GBC‐SD cell lines were analysed. (B) The efficiency of ADM siRNAs was validated by qRT‐PCR. (C) Western blot analysis was conducted to assess ADM knockdown efficiency in HuCCT1 and NOZ cells. (D) CCK8 assays were performed to evaluate cell proliferation in HuCCT1 and NOZ cells transfected with ADM siRNAs. (E) Colony formation assays were conducted to determine the effect of ADM knockdown on colony formation in HuCCT1 and NOZ cells. (F) Wound healing assays were used to evaluate migration in HuCCT1 and NOZ cells. (G) Transwell assays were performed to measure cell migration in HuCCT1 and NOZ cells transfected with ADM siRNAs; scale bar = 100 μm. *p < 0.05, **p < 0.01, ***p < 0.001.

To validate the effect of ADM on BTC progression, we first examined the expression of ADM in two ICC cell lines, HuCCT1 and RBE, human normal intrahepatic biliary epithelial cells (HIBEC), two GBC cell lines, NOZ and GBC‐SD, and a gallbladder epithelial cell line, HGEpC. We found that ADM expression was elevated in four cell lines: ICC and GBC. Consequently, the HuCCT1 and NOZ cell lines were chosen for subsequent experiments (Figure 3A). To elucidate the biological role of ADM in BTC, we conducted interference experiments to modulate ADM expression and observed its impact both in vivo and in vitro. siRNA targeting the splice junction was employed to knock down ADM expression. The effectiveness of the siRNA was verified through qRT‐PCR and WB (Figure 3B,C). The effect of ADM on cell proliferation was initially assessed using CCK‐8 and clone formation assays, with results indicating that ADM knockdown inhibited the proliferation of HuCCT1 and NOZ cells (Figure 3D,E). Additionally, ADM knockdown impeded cell migration as evidenced by wound healing and transwell assays (Figure 3F,G). These findings demonstrated the oncogenic role of ADM in ICC and GBC cells.

**FIGURE 4:**
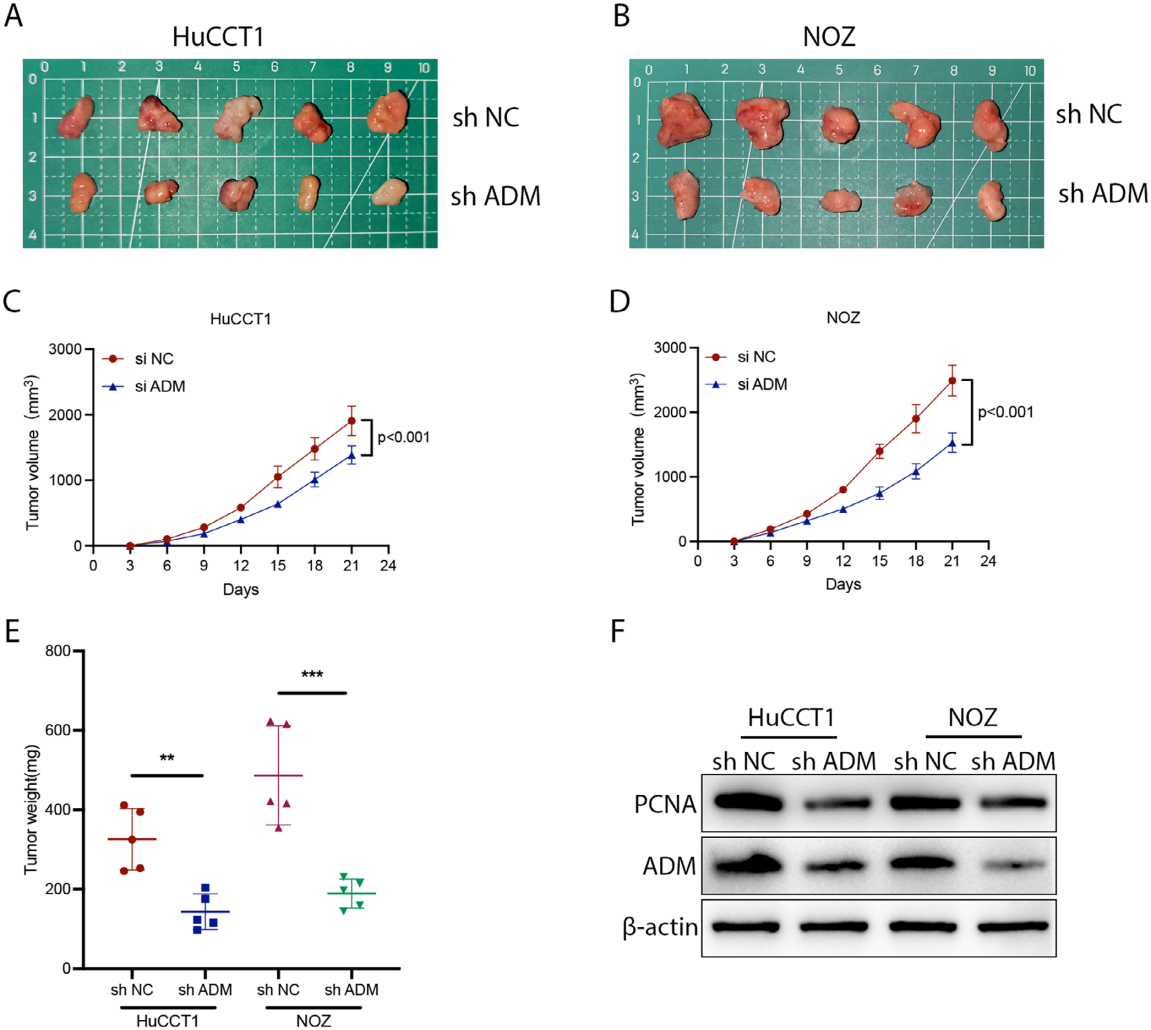
ADM promotes tumour growth in BTC in vivo. (A, B) Representative images of subcutaneous xenograft tumours (*n* = 5). (C, D) Tumour growth curves were plotted. (E) Tumour weight was measured after the mice were sacrificed and the tumours were resected. Compared to the lv‐Con groups, ADM knockdown significantly suppressed tumour growth in nude mice. *p < 0.05, **p < 0.01, ***p < 0.001. (F) ADM and PCNA protein expression levels in mouse subcutaneous xenograft tumour tissues.

ADM stabilisation‐deficient HuCCT1 and NOZ cell lines were constructed using lentiviruses containing shRNA against human ADM. These cells were then injected subcutaneously into nude mice and monitored over a 3‐week period. Tumour growth was significantly slower in ADM‐deficient cells compared to the control group (Figure 4C,D). Additionally, ADM‐deficient NOZ and GBC‐SD cells developed smaller tumours (Figure 4A,B,E). The proliferation marker proliferating cell nuclear antigen (PCNA) was also suppressed following ADM knockdown, as confirmed by Western Blot analysis (Figure 4F). These findings suggest that ADM plays a crucial role in promoting BTC development both in vitro and in vivo.

### 3.4 ADM Antagonist Inhibits the Proliferation and Migration of BTC In Vitro

**FIGURE 5:**
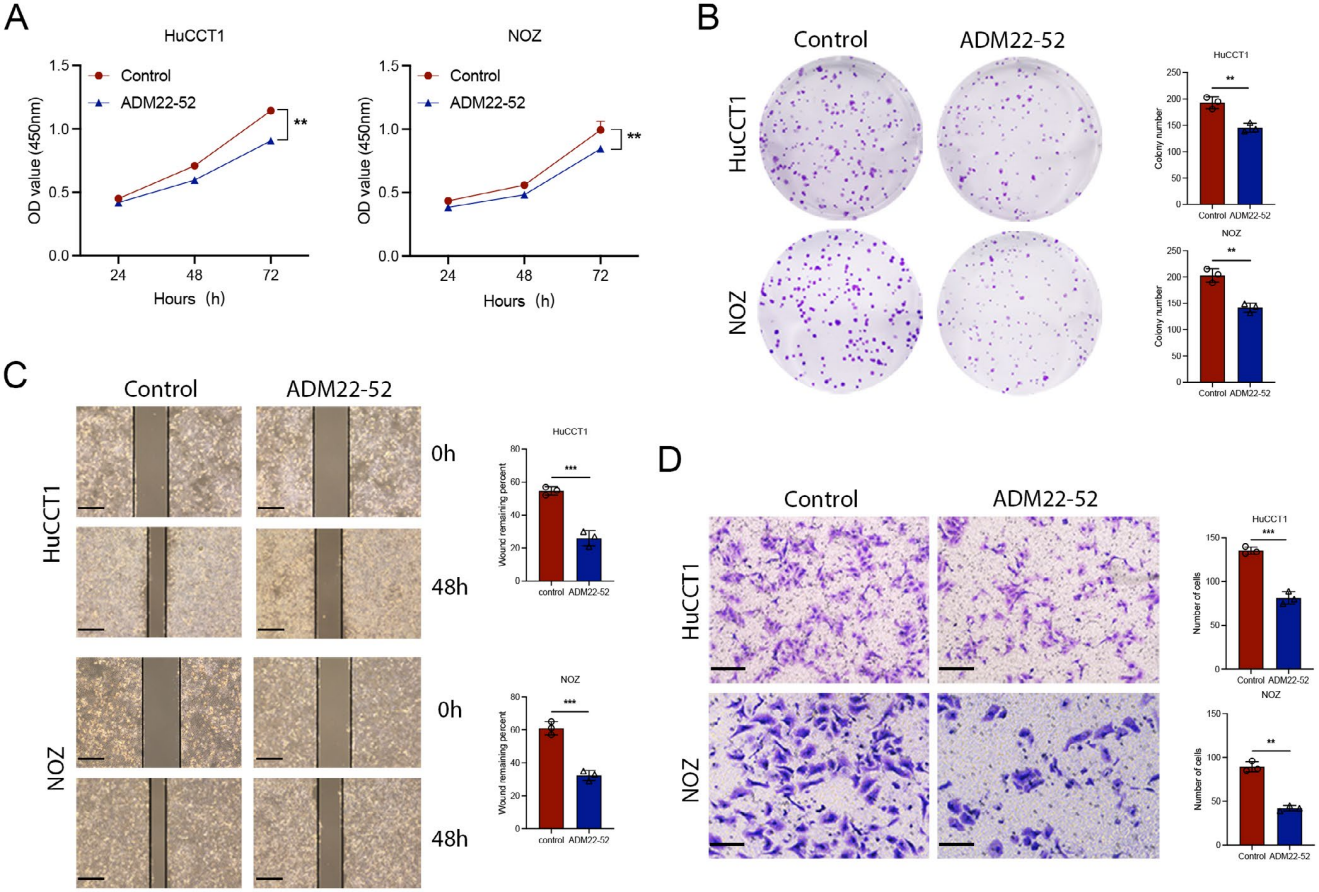
Adrenomedullin antagonist promotes the proliferation and migration of BTC cells in vitro. (A) CCK8 assays were conducted to assess cell proliferation in HuCCT1 and NOZ cells transfected with ADM22‐52. (B) Colony formation assays were performed on HuCCT1 and NOZ cells to evaluate their ability to form colonies. (C) Wound healing assays were utilised to assess the migration capabilities of HuCCT1 and NOZ cells. (D) Transwell assays were conducted on HuCCT1 and NOZ cells supplemented with ADM22‐52 to measure cell migration. scale bar = 100 μm. scale bar = 100 μm. *p < 0.05, **p < 0.01, ***p < 0.001.

To investigate the effect of adrenomedullin antagonists on the cell viability and proliferation of BTC, HuCCT1 and NOZ cells were treated with varying concentrations of ADM22‐52 for 48 h. CCK‐8 assay results revealed that the inhibitory effect on BTC cells became more pronounced as the concentration of ADM22‐52 increased (Figures S2A and S2B). It was observed that a 1 nM concentration of ADM22‐52 significantly suppressed the viability of both cell lines after 48 h of treatment. Therefore, a concentration of 1 nM ADM22‐52 was selected for subsequent experiments. Initially, the impact of ADM22‐25 on cell proliferation was assessed using the CCK‐8 assay and colony formation assay (Figure 5A,B). The results demonstrated that the exogenous addition of ADM22‐25 inhibited the proliferation of HuCCT1 and NOZ cells. Furthermore, the exogenous addition of ADM22‐25 impaired the migration ability of these cells, as evaluated by the wound healing and transwell assays (Figure 5C,D). These findings illustrate the inhibitory effect of ADM22‐25 on the proliferation and migration of BTC cells.

### 3.5 ADM Promotes Biology Function of HUVECs via Coordinating With VEGF to Enhance Akt Phosphorylation

**FIGURE 6:**
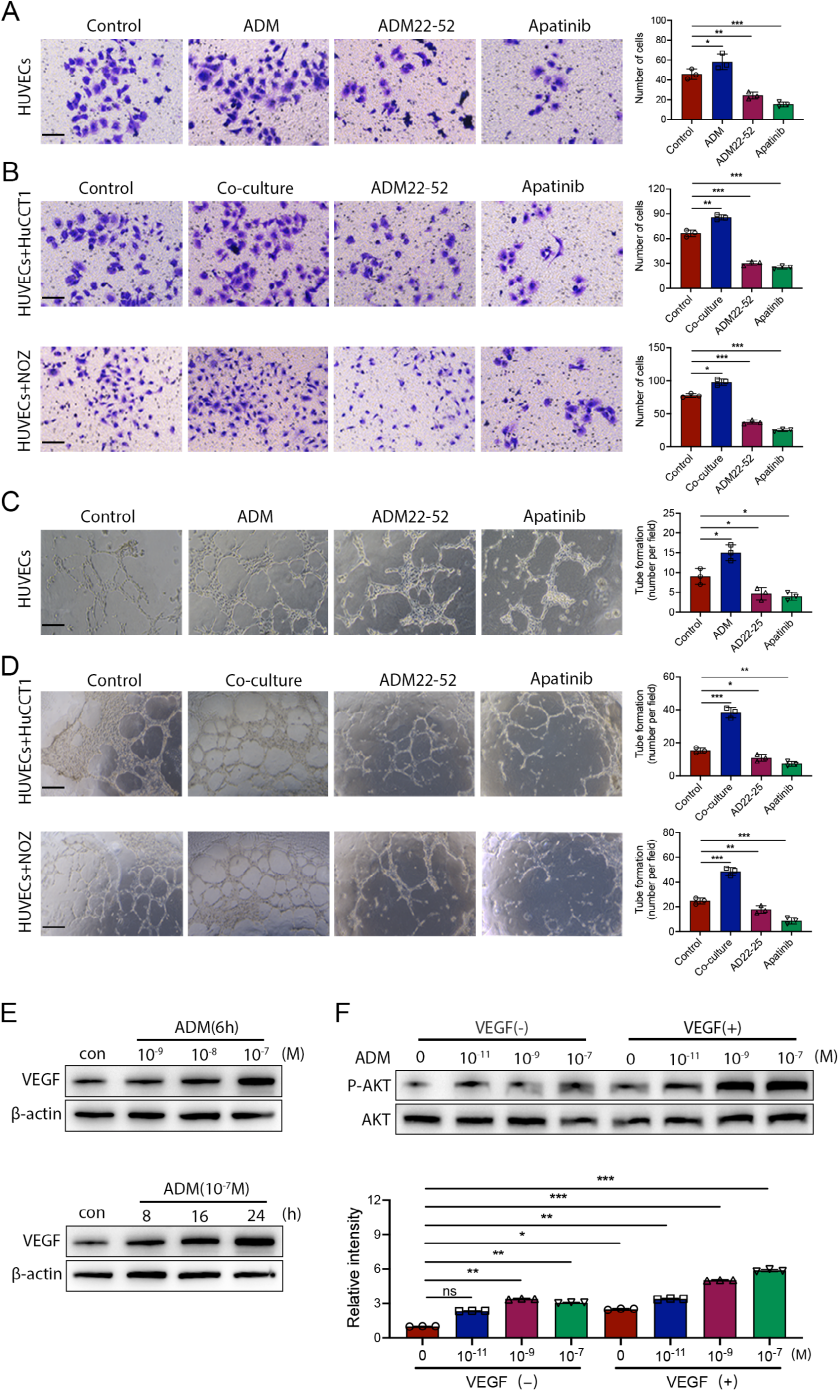
The effects of ADM, ADM22‐52, and Apatinib on HUVEC migration and tube formation were assessed in vitro. (A) HUVECs were cultured on transwells with ADM (100 nM), ADM22‐52 (1 nM) or Apatinib (2 μM) for 24 h, with PBS serving as the control, scale bar = 100 μm. (B) HUVECs were placed in the upper chamber of the transwell and co‐cultured with HuCCT1 or NOZ cells in the lower chamber, receiving treatments with ADM (100 nM), ADM22‐52 (1 nM) or Apatinib (2 μM) for 24 h, scale bar = 100 μm. (C) HUVECs were seeded on Matrigel and treated with ADM (100 nM), ADM22‐52 (1 nM) or Apatinib (2 μM) for 24 h to evaluate tube formation, scale bar = 100 μm. (D) HUVECs were inoculated onto Matrigel and co‐cultured with HuCCT1 or NOZ cells, receiving treatments with ADM (100 nM), ADM22‐52 (1 nM) or Apatinib (2 μM) for 24 h, scale bar = 100 μm. (E) VEGF expression in human HUVEC cells was upregulated in a dose‐dependent and time‐dependent manner by ADM administration. (F) The analysis of Akt and p‐Akt levels in HUVECs treated with ADM (10^−11^ to 10^−7^ mol/L) alone or with ADM + VEGF (10 ng/mL) showed that ADM stimulated Akt pathway activation in a dose‐dependent manner. The histogram displays the relative expression intensity of p‐Akt, with red and blue bars representing ADM alone and ADM + VEGF, respectively.

Vascular abnormalities are a hallmark of most solid tumours and facilitate immune evasion, inhibit the efficacy of immunotherapy [15]. Thus, combining antiangiogenic therapies and immunotherapies might increase the effectiveness of immunotherapy. In order to determine the direct angiogenic capability of ADM, we investigated various angiogenic properties of human umbilical vein endothelial cells (HUVECs). In addition to HuCCT1 and NOZ cells, the effects of ADM, ADM22‐52 and Apatinib on HUVECs were also evaluated. HUVECs were treated with different concentrations of ADM, ADM22‐52 and Apatinib for 48 h, and cell viability was assessed using the CCK‐8 assay. The results indicated that ADM, ADM22‐52 and Apatinib inhibited HUVEC proliferation in a dose‐dependent manner (Figure S3A–S3C). Significant inhibition of HUVEC viability was observed after 48 h of treatment with 100 nM ADM, 1 nM ADM22‐52 and 2 μM Apatinib. Therefore, these concentrations were selected for subsequent experiments. A perforated chamber system was employed to assess the impact of ADM on HUVECs migration. Initially, four experimental groups were established to study the migratory capacity of HUVECs under different conditions: a control group, exogenous ADM addition (100 nM, 24 h), exogenous ADM22‐25 addition (1 nM, 24 h) or co‐cultivation with HuCCT1/NOZ, and exogenous Apatinib addition (2 μM, 24 h). The results demonstrated that exogenous ADM significantly promoted HUVECs migration. The migratory capacity of HUVECs co‐cultivated with HuCCT1/NOZ cells was higher than that of HUVECs cultured alone. Conversely, ADM22‐25 and Apatinib significantly inhibited HUVECs migration. Consistently, ADM22‐52 also reduced the migratory potential of HUVECs co‐cultivated with HuCCT1/NOZ cells (Figure 6A,C).

Furthermore, we evaluated the effect of ADM on capillary‐like structure formation by culturing HUVECs on Matrigel using the same treatment methods. The results showed that the exogenous addition of ADM enhanced the ability of cultured HUVECs to form capillary‐like structures. HUVECs co‐cultured with HuCCT1/NOZ cells exhibited a higher tube‐forming capacity compared to those cultured alone, whereas ADM22‐25 and Apatinib significantly inhibited HUVEC tube formation (Figure 6B,D). Our study demonstrates that exogenous ADM significantly promotes HUVECs proliferation, migration and tube formation, and that ADM secreted by HuCCT1 and NOZ cells is crucial for HUVEC proliferation, migration and capillary‐like structure formation.

Research indicates that ADM promotes tumour angiogenesis by upregulating the expression of VEGF. Upon activation of its receptors, VEGF further initiates the AKT signalling pathway [16]. As a crucial downstream molecule, AKT plays significant roles in various cellular processes, including proliferation, survival and metabolism [17]. Strikingly, we found that ADM administration increased VEGF expression in a dose‐ and time‐dependent manner (Figure 6E). Additionally, activation of the Akt pathway was analysed in endothelial cells by evaluating levels of phospho‐Akt (p‐Akt). It was found that, although ADM alone elicited minimal activation of the pathway, in the presence of VEGF (10 ng/mL), ADM (10^−11^ to 10^−7^ mol/L) dose‐dependently increased levels of p‐Akt (Figure 6F).

### 3.6 ADM Activates CRLR‐VE‐Cadherin Signalling to Destabilise Endothelial Adherens Junctions

**FIGURE 7:**
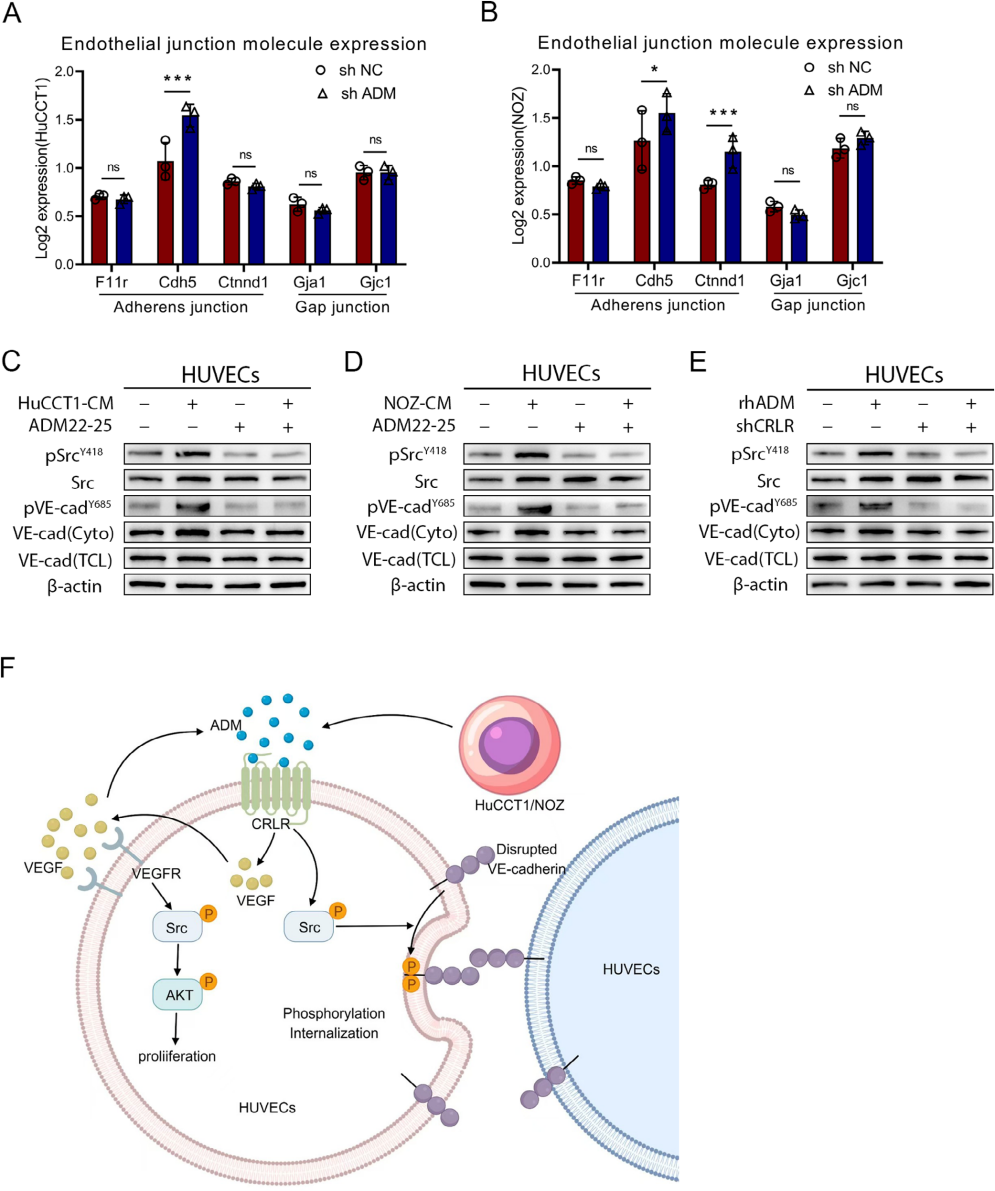
ADM activates CRLR‐VE‐cadherin signalling. (A, B) A violin plots depict the expression levels of cell junction molecules in sh NC and sh ADM endothelial cells (ECs). (C–E) Western blot analysis reveals the expression and phosphorylation status of Src, VE‐cadherin and their phosphorylated forms in treated HUVECs. Abbreviations: Cyto, Cytoplasm; TCL, Total Cell Lysate. (F) The diagram illustrates that ADM secreted by BTC cells interacts with CRLR on endothelial cells (ECs). This interaction disrupts the physical structure of ECs, impairing cell adhesion junctions through the activation of the p‐SRC signalling pathway. Additionally, it promotes ECs proliferation via the VEGF/VEGFR/AKT signalling pathway.

Previous studies have demonstrated that ADM secreted by hypoxia‐TAM can activate CRLR signalling in endothelial cells (ECs), thereby disrupting vascular adhesion protein‐mediated adhesion junctions, leading to the dissociation of endothelial connections and the internalisation of VE‐cadherin [14]. Multiplex immunohistochemical (mIHC) staining revealed that in non‐responding biliary tract cancer (BTC) tissues treated with targeted and immune combination therapy, there was an increase in ADM and CD31 expression, a decrease in VE‐cadherin levels, an increase in vascular density and disrupted vascular morphology (Figures S4A and S4B). mIHC staining of CD4+ and CD8+ T cells in human BTC tissues (Figure S4C). Experiments showed that compared to the control, shADM‐derived HuCCT1 and NOZ cells showed enrichment in the adhesion junction pathway and increased expression of genes encoding VE‐cadherin (Figure 7A,B). To determine how tumour cell‐secreted ADM damages endothelial adhesion junctions, HuCCT1 or NOZ tumour supernatant and ADM22‐52 were used to treat HUVECs. Under the stimulation of HuCCT1 and NOZ tumour supernatant, the phosphorylation of Src and VE‐cadherin and the subsequent internalisation of VE‐cadherin were significantly increased, whereas treatment with ADM22‐25 greatly inhibited this signalling activation (Figure 7C,D). Similarly, silencing CRLR in HUVECs using shRNA also significantly affected rhADM‐induced phosphorylation of Src and VE‐cadherin (Figure 7E). These results suggest that ADM secreted by BTC cells interacts with CRLR on endothelial cells (ECs). This interaction disrupts the physical structure of ECs, impairing cell adhesion junctions through the activation of the p‐SRC signalling pathway. Additionally, it promotes ECs proliferation via the VEGF/VEGFR/AKT signalling pathway (Figure 7F).

### 3.7 Microenvironmental Landscape in BTC Patients With High and Low Adrenomedullin Expression

**FIGURE 8:**
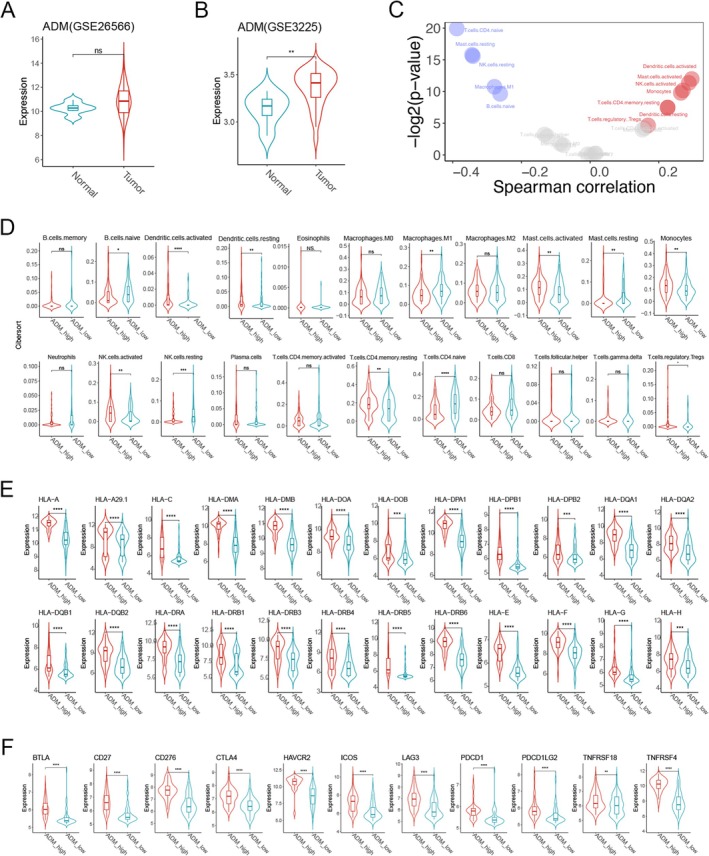
The immune landscape of ADM‐high and ADM‐low expression groups. (A, B) Violin plots show the median and quartile estimations of ADM expression levels in ICC patients and normal samples; (C) Immune cell infiltration in ADM‐high and ADM‐low groups; (D) Box plots display 22 immune cells between different subtypes; (E, F) Box plots present differential expression of HLA genes (E) and multiple immune checkpoints (F) between ADM‐high and ADM‐low groups. ADM, adrenomedullin; HLA, human leukocyte antigen. *p < 0.05, **p < 0.01, ***p < 0.001, ****p < 0.0001.

To further elucidate the impact of ADM on immunotherapy, we initially analysed the correlation between ADM expression and immune cell infiltration. The results indicated that in the GSE26566 and GSE3225 datasets, ADM expression was higher in cancerous tissues compared to normal ICC tissues (Figure 8A,B). Then, we utilised TIMER combined with the LM22 signature matrix to assess the differences in tumour‐infiltrating immune cells between the ADM high‐expression and low‐expression groups. Figure 8C shows the results of ICC patients and normal samples from the TCGA cohort. As shown in Figure 8D, ICD‐high subtype patients exhibited an increased proportion of memory B cells, dendritic cells, M1 macrophages, mast cells, monocytes, NK cells, activated memory CD4+ cells and Tregs. The infiltration of immune cells plays a vital role in immunotherapy, and the exhaustion of immune checkpoints is also a significant aspect of its effectiveness. mIHC staining revealed that, compared to BTC patient samples with no response to camrelizumab combined with apatinib treatment, tumour tissues from responders exhibited significantly higher levels of CD4+ and CD8+ T cell infiltration (Figure S4D). Additionally, most of the immune checkpoints and the human leukocyte antigen (HLA) genes were highly expressed in the ADM high‐expression group. In contrast, the ADM low‐expression group exhibited the opposite results (Figure 8E,F). Together, these results demonstrated that the ADM high‐expression group was related to the active immune phenotype, while the ADM low‐expression group was related to the suppressive immune phenotype.

## 4 Discussion

Gemcitabine and cisplatin chemotherapy has long served as the standard first‐line treatment for patients with locally advanced or metastatic BTC [18]. However, recent advances in immunotherapy have sparked a paradigm shift [19, 20]. Vascular abnormalities and immunosuppression are prominent features in most solid tumours, contributing to structural and functional irregularities in tumour blood vessels that hinder immune effector cell recruitment [21]. This limitation compromises the efficacy of cancer immunotherapies. The interplay between vascular normalisation and immune reprogramming presents a unique opportunity to explore novel combination therapy strategies aimed at enhancing antitumour immunity.

Thus, we use immune checkpoint inhibitors and anti‐angiogenic drug medication strategies, which employ camrelizumab in combination with apatinib, in patients with advanced BTC, revealing notable antitumor activity with an overall response rate (ORR) of 68.4% and an acceptable safety profile, aligning with findings from prior research [22]. The one‐year relapse rate for advanced BTC patients is approximately 60%. Additionally, Mauro E [23] and Schirizzi A [24] reported one‐year recurrence rates of 66% and 57%, respectively, despite undergoing radical surgery. In this study, the one‐year recurrence rates were 53.8% for patients who responded to treatment, 83.3% for those who did not respond and 63.1% for all patients with biliary tract cancer (BTC). Despite the lack of a significant reduction in the one‐year recurrence rate compared to previous studies, the research team was encouraged by the outcomes. Notably, the overall survival (OS) for two patients exceeded 40.0 months, with recorded durations of 40.3 and 60.0 months, and the progression‐free survival (PFS) for these patients was 27.0 and 17.9 months, respectively. Although the combination of apatinib and camrelizumab demonstrated good clinical efficacy in advanced BTC, some patients exhibited poor responses to this therapy. The mechanisms responsible for this variability in efficacy are not yet fully understood. Therefore, identifying reliable tumour biomarkers for early detection and targeted molecular therapy is imperative.

Subsequently, we investigated transcriptomic profiles in clinical responses to the combination of apatinib and camrelizumab in locally advanced BTC. Our findings highlight the significant association of ADM with prognosis and drug resistance in BTC patients. ADM, a peptide involved in various physiological processes including vascular health and hormone regulation, has been implicated in cancer progression by promoting proliferation, angiogenesis and metastasis [25]. In our study, RT‐qPCR, WB and IHC experiments revealed significantly upregulated ADM expression in both responder and non‐responder groups. ADM was identified as a tumour promoter in BTC, exhibiting higher expression compared to normal tissues. In vitro and in vivo experiments further demonstrated that ADM enhances BTC cell proliferation, clonogenicity and migration. Importantly, ADM inhibitors such as ADM22‐25 effectively suppressed BTC cell proliferation, aligning with endogenous ADM interference results. In brief, we demonstrated that ADM plays a crucial role in BTC progression.

Numerous comprehensive studies have underscored the pivotal role of AD in drug resistance across various cancers. ADM enhances resistance to sunitinib in clear cell renal cell carcinoma by downregulating FDX1 expression and inhibiting cuproptosis [26]. Furthermore, ADM promotes chemoresistance to cisplatin in ovarian cancer [27]. Additionally, the ADM‐CRLR axis regulates drug‐tolerant, relapse‐initiating cells in acute myeloid leukaemia [28]. ADM functions as an autocrine/paracrine growth factor promoting angiogenesis and vascular abnormalities, which hinder immune effector cell infiltration and activation, thereby complementing cancer immunotherapy strategies. In our investigation, we directly examined ADM's effects on angiogenesis by treating HUVECs with ADM, ADM22‐52 and apatinib. ADM stimulated HUVEC proliferation, migration and tube formation, while ADM22‐52 and apatinib countered these effects. Similar outcomes were observed in a co‐culture system of HUVECs and BTC cells, aligning with previous findings [27]. Angiogenesis, a complex process regulated by classic factors like VEGF, HIF‐1α and FGF‐2, serves as a primary target for anti‐angiogenic therapies [28]. Our study demonstrated that ADM upregulates VEGF expression in endothelial cells in a dose‐ and time‐dependent manner. Moreover, ADM enhances VEGF‐mediated activation of the Akt pathway, crucial for angiogenesis [29]. In contrast, apatinib inhibits tumour angiogenesis by disrupting VEGF‐VEGFR2 interaction, thereby suppressing BTC cells proliferation. However, ADM's direct effects on BTC vasculature integrity remain poorly understood. To further explore the potential reasons for resistance to combined immunotherapy and anti‐angiogenesis treatment, we conducted an analysis of the immune microenvironment in BTC. The results indicated that compared to the low ADM expression group, the high ADM expression group exhibited a more significant enrichment of tumour‐infiltrating immune cells, along with an upregulation of immune checkpoint expression. Our findings indicate that elevated levels of ADM lead to the internalisation of VE‐cadherin, which destabilises vascular endothelial junctions. This process enhances tumour vasculature permeability, potentially compromising the efficacy of immune‐combination anti‐vascular treatments.

BTC is a relatively rare and highly heterogeneous malignancy. This study, as a single‐center exploratory investigation, is the first to evaluate the efficacy and safety of apatinib combined with camrelizumab in advanced BTC patients, while also analysing associated biomarkers, offering significant clinical insights. However, the study has several limitations: (1) The limited number of BTC patients may impact the multivariate analysis results and introduce bias; (2) The lack of a control group and external validation cohort may reduce the generalisability and persuasiveness of the findings; (3) Tumour microenvironmental disparities between ADM‐high and ADM‐low BTC subtypes were exclusively extrapolated from curated public repositories, without experimental validation through independent sequencing. Currently, most research on targeted and immunologic combination therapies targeting vascular components in BTC remains exploratory, with no Phase III clinical data available. To address these limitations, a detailed future research plan has been developed: first, additional patients will be enrolled at our institution to evaluate the impact of ADM on the efficacy of targeted immunotherapy; second, collaboration with multi‐center research teams will be sought to validate clinical results in diverse patient populations across different regions. Moreover, to further explore the underlying mechanisms of the causal relationship between ADM and treatment resistance, an ICCA plasmid‐induced mouse orthotopic tumour model has been successfully established. Following model establishment, the effects of combined immune and targeted therapies using ADM and VEGF inhibitors will be investigated in vivo. In fact, pharmacological perturbation of ADM stabilises endothelial junctions in tumour vasculature but exhibits minimal effects on blood vessels in the normal brain, indicating that ADM is a selective and druggable target [30]. These validation efforts are expected to significantly strengthen the credibility of the study, provide a solid theoretical foundation for clinical translation, and lay the groundwork for more comprehensive scientific trials.

Our study underscores the role of ADM in mediating drug resistance to the combination of apatinib and camrelizumab through several mechanisms: (1) direct promotion of BTC cells proliferation and survival; (2) induction of vascular abnormalities that hinder immune cell infiltration and drug delivery; (3) transformation of cell phenotypes towards a more aggressive state. These findings emphasise the critical need for developing strategies that target ADM to mitigate tumour progression and enhance tumour responsiveness to immunotherapy and anti‐vascular therapy.

## Author Contributions

**Zhengfeng Xuan:** conceptualization (equal), data curation (equal), investigation (equal), methodology (equal), project administration (equal), software (equal), validation (equal), writing – original draft (lead), writing – review and editing (equal). **Haoran Hu:** conceptualization (equal), data curation (equal), investigation (equal), methodology (equal), project administration (equal), validation (equal), writing – original draft (equal), writing – review and editing (equal). **Jian Xu:** conceptualization (equal), data curation (equal), investigation (equal), methodology (equal), project administration (equal), validation (equal), writing – original draft (equal), writing – review and editing (equal). **Xiaowei Ling:** data curation (supporting), investigation (equal), supervision (equal), validation (supporting), writing – review and editing (supporting). **Long Zhang:** data curation (supporting), investigation (equal), supervision (equal), validation (supporting), writing – review and editing (supporting). **Wenzhu Li:** data curation (supporting), investigation (equal), supervision (equal), validation (supporting), writing – review and editing (supporting). **Junda Li:** data curation (supporting), investigation (equal), supervision (equal), validation (supporting), writing – review and editing (supporting). **Chan Zhu:** data curation (supporting), formal analysis (equal), methodology (supporting), software (equal), supervision (equal), validation (supporting), writing – review and editing (supporting). **Yunjie Song:** data curation (supporting), formal analysis (equal), methodology (equal), software (equal), supervision (equal), validation (supporting), writing – review and editing (supporting). **Xing Zhang:** data curation (supporting), formal analysis (equal), methodology (supporting), software (equal), supervision (equal), validation (supporting), writing – review and editing (equal). **Jianhua Rao:** conceptualization (lead), formal analysis (equal), funding acquisition (equal), methodology (equal), project administration (equal), resources (equal), supervision (lead), writing – original draft (equal), writing – review and editing (lead). **Yong Wang:** conceptualization (lead), formal analysis (lead), funding acquisition (equal), methodology (equal), project administration (equal), resources (lead), supervision (lead), writing – original draft (equal), writing – review and editing (lead). **Feng Cheng:** conceptualization (lead), formal analysis (lead), funding acquisition (lead), methodology (equal), project administration (lead), resources (lead), supervision (lead), writing – original draft (equal), writing – review and editing (lead).

## Ethics Statement

Ethics Committee of the First Affiliated Hospital of Nanjing Medical University. Ethical approval number: 2023‐SR‐130.

## Consent

Participants gave informed consent to participate in the study before participating.

## Conflicts of Interest

The authors declare no conflicts of interest.

## Supporting information


Data S1

Data S2

## Data Availability

All data generated from this study, including Supporting Information and Raw data, are available from the corresponding author on reasonable request.
