# Supplementary material for: Adrenomedullin Inhibits the Efficacy of Combined Immunotherapy and Targeted Therapy in Biliary Tract Cancer by Disrupting Endothelial Cell Functions
Source: J Cell Mol Med. 2025 Mar 12;29(5):e70460. doi: 10.1111/jcmm.70460 (PMC11903196; doi:10.1111/jcmm.70460)
Supplement: Supplementary file 1 — Data S1 [file JCMM-29-e70460-s001.docx]

**Adrenomedullin inhibits the efficacy of combined immunotherapy and targeted therapy in biliary tract cancer by disrupting endothelial cell functions**

**Supplementary Figure 1**

**Supplementary Figure 2**

**Supplementary Figure 3**

**Supplementary Figure 4**

**Supplementary Table 1**

**Supplementary Table 2**

**Supplementary Table 3**


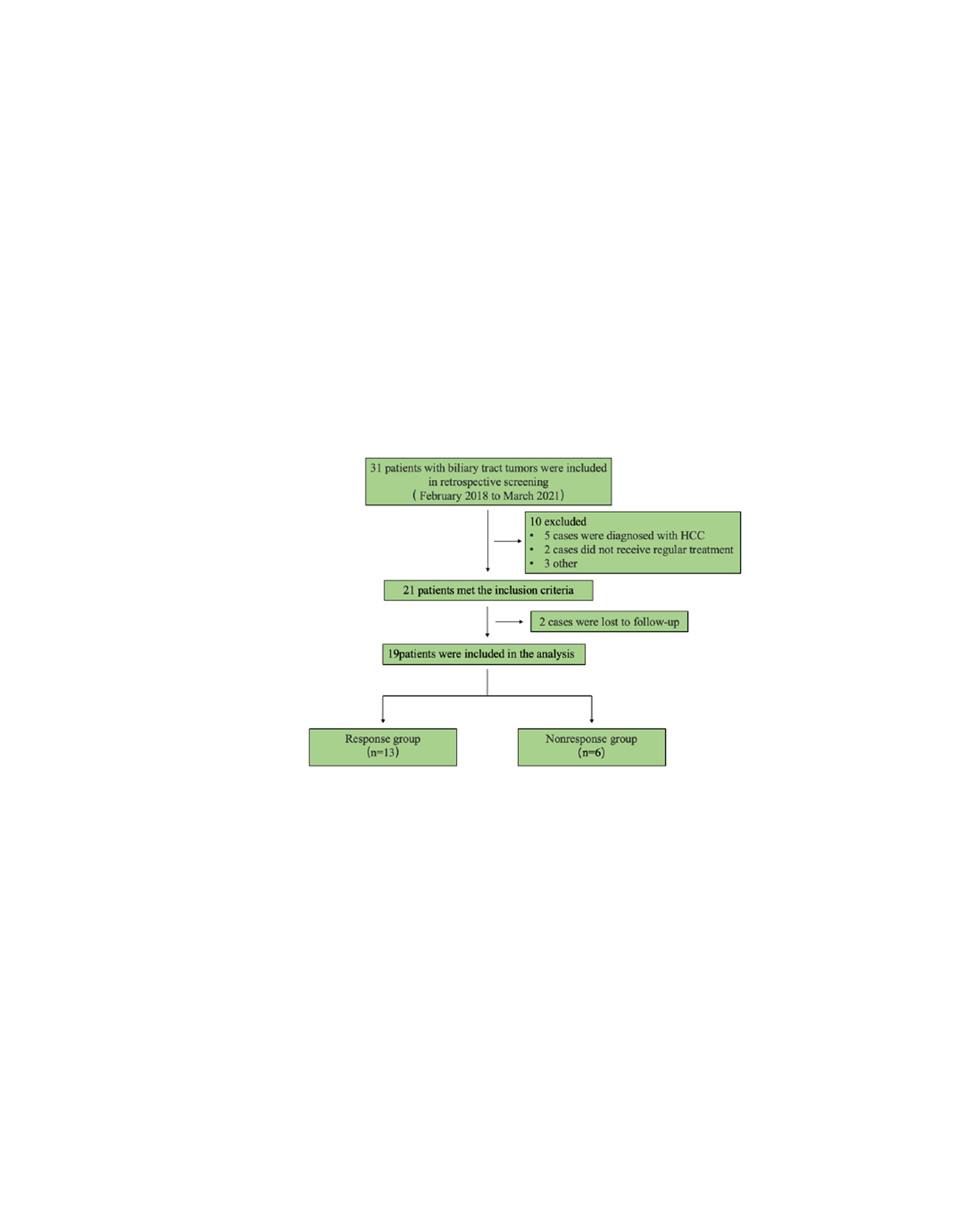


**Supplementary Figure 1:** Patient selection flowchart

**
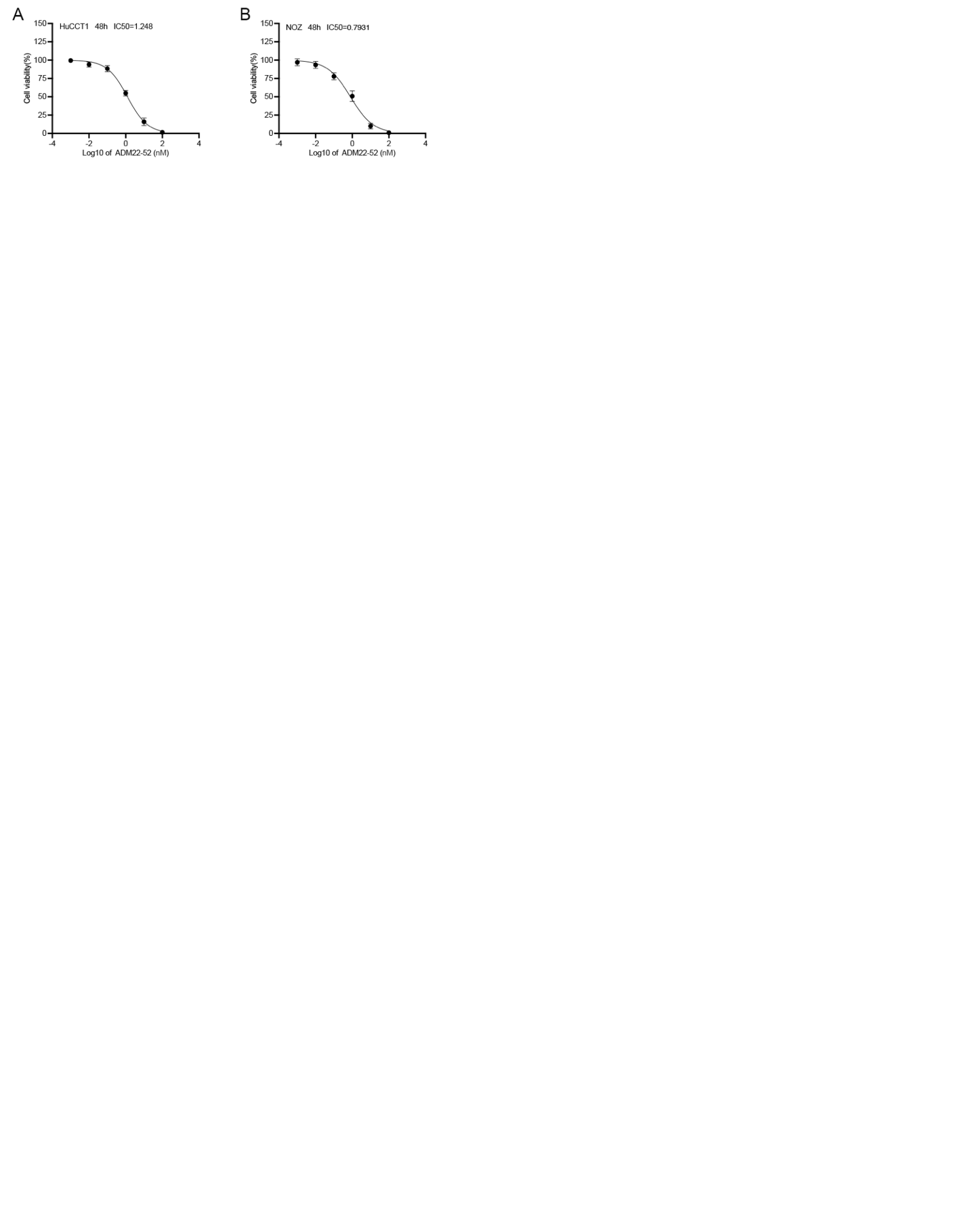
**

**Supplementary Figure 2.** A and B show proliferation curves of HuCCT1 and NOZ cell lines following treatment with graded concentrations of ADM22-52.


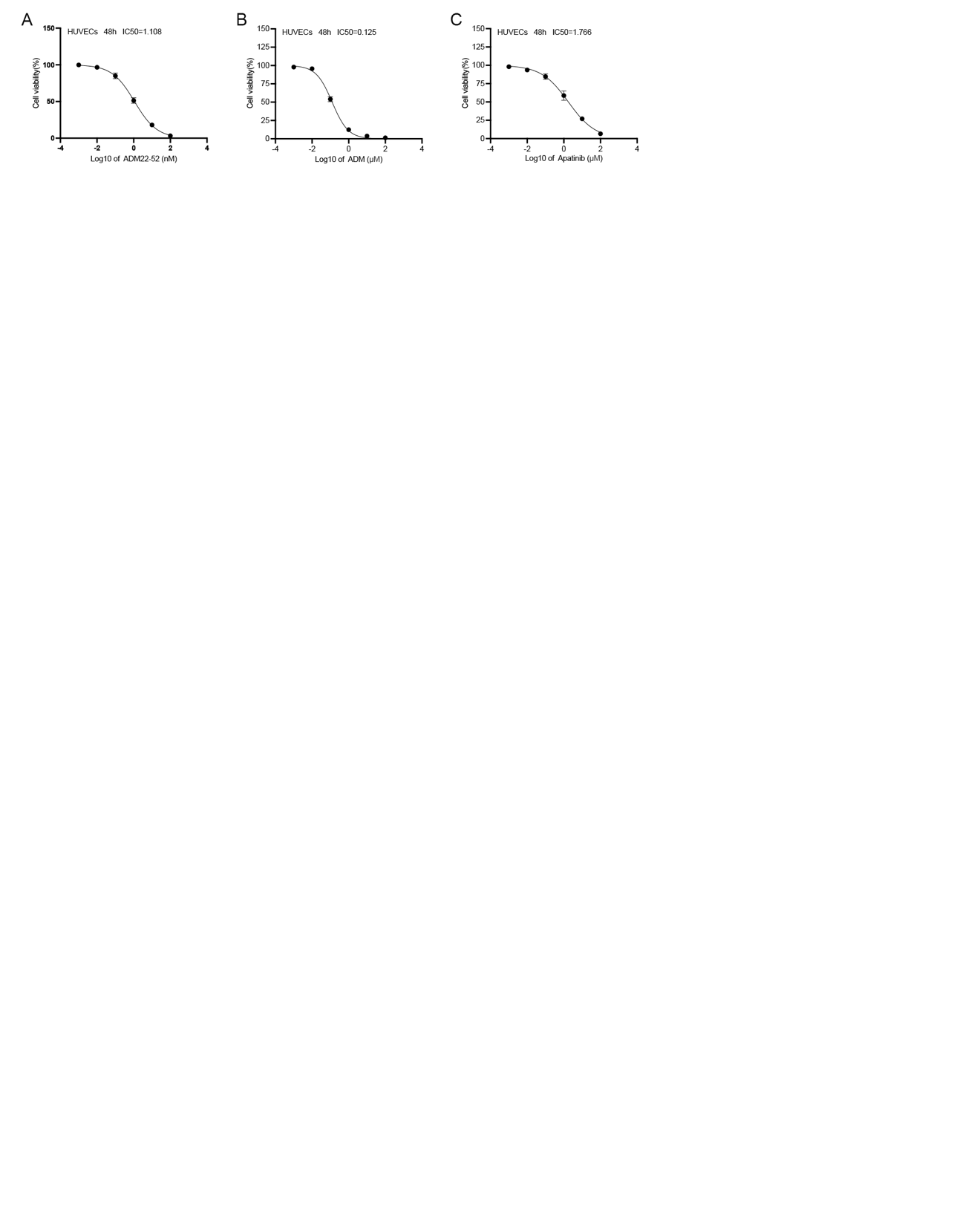


**Supplementary Figure 3.** A-C, proliferation curves of HUVECs were constructed following treatment with varying concentrations of Apatinib (A), ADM (B), and ADM22-52 (C).

**
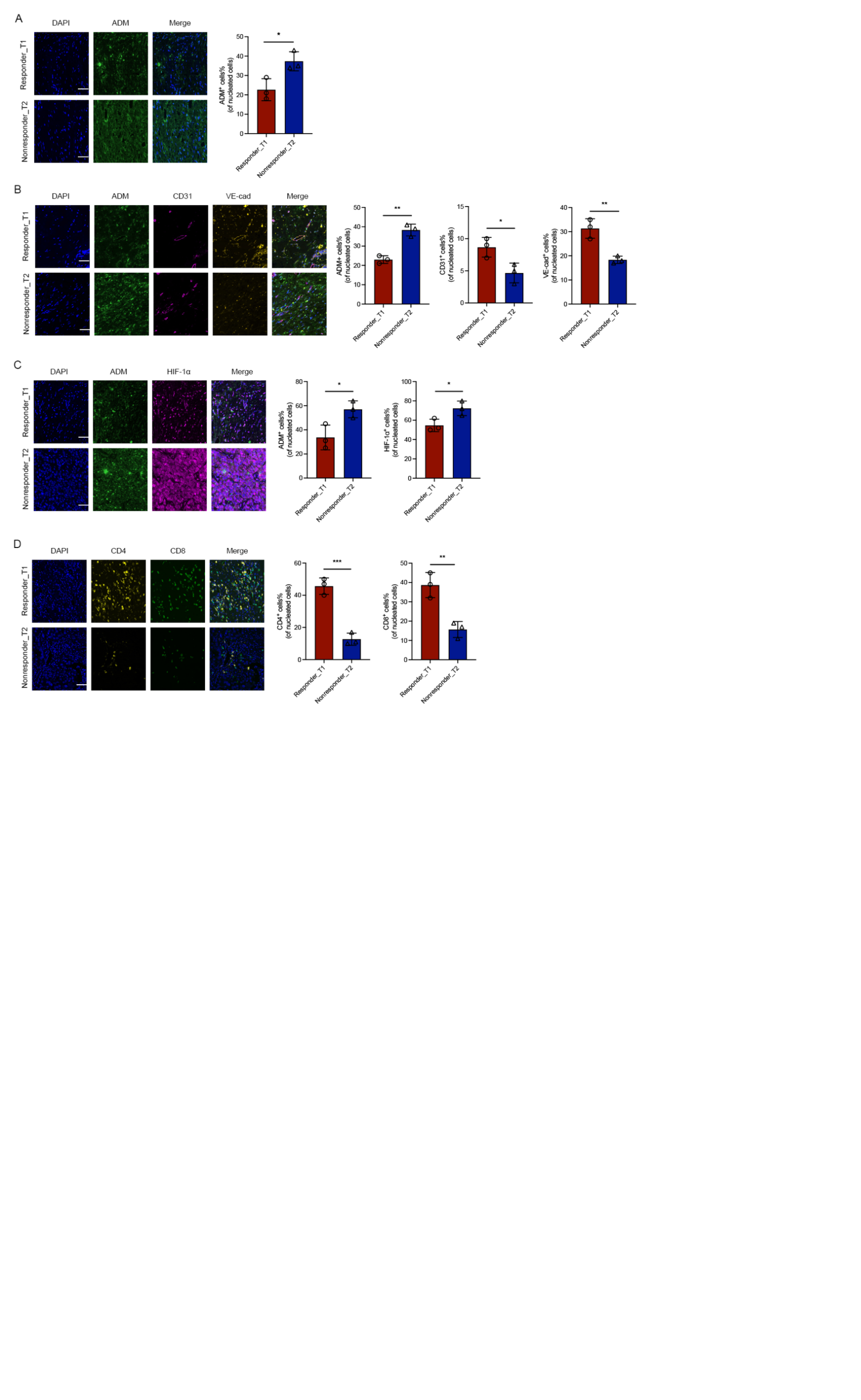
**

**Supplementary Figure 4.** (A and B) mIHC staining of ADM (A), CD31 and VE-cadherin (B) in human BTC tissues. (C) mIHC staining of ADM and HIF-1α in human BTC tissues. (D) mIHC staining of CD4+ and CD8+ T cells in human BTC tissues. Scale bar: 50 µm. *P < 0.05, **P < 0.01, ***P < 0.001, ****P < 0.0001. mIHC staining of CD4+ and CD8+ T cells in human BTC tissues.

**Supplementary Table 1.** Baseline characteristics of patients with local advanced biliary tract cancer treated with camrelizumab plus apatinib

| Parameter | Result, n (%) |
| --- | --- |
| **Age (years), n (%)** |  |
| <65 | 12 (63.2%) |
| ≥65 | 7 (36.8%) |
| **Sex, n (%)** |  |
| Male | 12 (63.2%) |
| Female | 7 (36.8%) |
| **Median age at diagnosis, yr (range)** | 61.0 (38-79) |
| **Histology, n (%)** |  |
| GBC | 11 (57.9%) |
| ICC | 8 (42.1%) |
| **ECOG PS, n (%)** |  |
| 0 | 5 (26.3%) |
| 1 | 7 (36.8%) |
| 2 | 7 (36.8%) |
| **Sites of metastases, n (%)** | |
| Lymph nodes | 8 (42.1%) |
| blood vessel | 2 (31.6%) |
| other | 8 (42.1%) |

**Supplementary Table 2.** The detailed baseline characteristics of the patients


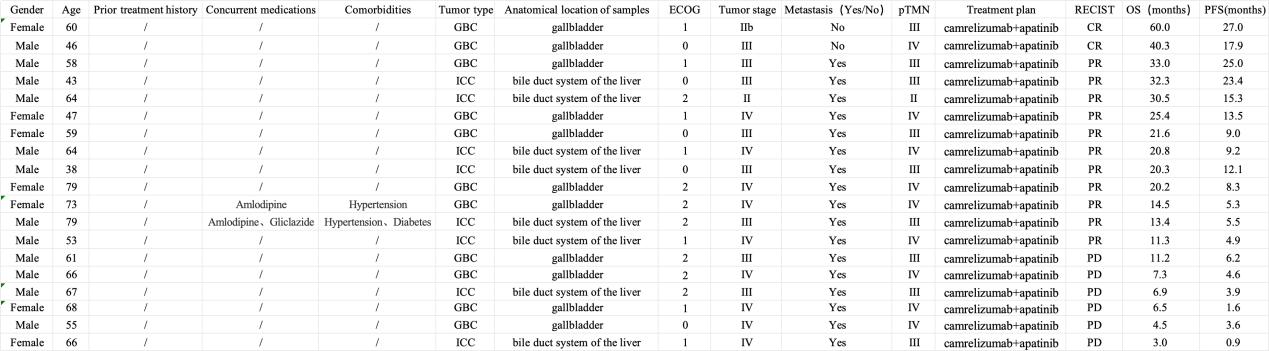


**Supplementary Table 3.** TME 289 gene list

| ABCF1 | CD44 | FCGR1A | IL21R | NFKBIA | TIE1 |
| --- | --- | --- | --- | --- | --- |
| ADM | CD47 | FCGR2B | IL2RA | NKG7 | TIGIT |
| ADORA2A | CD48 | FCRL2 | IL2RB | NOS2 | TLR3 |
| AKT1 | CD6 | FGF13 | IL2RG | NT5E | TLR7 |
| ANGPT2 | CD68 | FOXP3 | IL4 | OAS1 | TLR8 |
| ARG1 | CD69 | FPR1 | IL6 | OAS2 | TLR9 |
| ATM | CD70 | FUT4 | IL7R | OAS3 | TNF |
| AXL | CD74 | G6PD | IRF1 | PDCD1 | TNFRSF14 |
| BCL2 | CD79A | GBP1 | IRF4 | PDCD1LG2 | TNFRSF17 |
| BIRC5 | CD79B | GNLY | IRF9 | PDGFA | TNFRSF18 |
| BLK | CD80 | GUSB | ISG15 | PDGFB | TNFRSF1A |
| BLM | CD84 | GZMA | ITGA1 | PECAM1 | TNFRSF1B |
| BRCA1 | CD86 | GZMB | ITGAE | PIK3CA | TNFRSF4 |
| BRCA2 | CD8A | GZMH | ITGAL | PIK3CD | TNFRSF9 |
| BRIP1 | CD8B | GZMK | ITGAM | PMS2 | TNFSF10 |
| BTLA | CDKN2A | HAVCR2 | ITGAX | PNOC | TNFSF13B |
| C1QA | CEACAM3 | HDC | ITGB2 | POLR2A | TNFSF18 |
| C1QB | CMKLR1 | HERC6 | KIR2DL3 | PRF1 | TNFSF4 |
| CCL13 | CPA3 | HIF1A | KIR3DL1 | PSMB10 | TNFSF9 |
| CCL18 | CSF1R | HLA-DMA | KIR3DL2 | PSMB9 | TRAT1 |
| CCL2 | CSF2 | HLA-DMB | KLRB1 | PTEN | TWIST1 |
| CCL20 | CSF2RB | HLA-DOA | KLRD1 | PTGER4 | VCAM1 |
| CCL21 | CSF3R | HLA-DOB | KLRK1 | PTGS2 | VEGFA |
| CCL22 | CTAG1B | HLA-DPA1 | LAG3 | PTPN11 | VTCN1 |
| CCL4 | CTLA4 | HLA-DQA2 | LCK | PTPRC | ZAP70 |
| CCL5 | CTSS | HLA-DRA | LILRB2 | PVR | ZEB1 |
| CCL7 | CTSW | HSD11B1 | LY9 | RAD51 | CXCL2 |
| CCND1 | CX3CL1 | ICAM1 | LYZ | RB1 | FCGR3B |
| CCR2 | CX3CR1 | ICOS | MAGEA1 | RORC | GZMM |
| CCR4 | CXCL1 | ICOSLG | MAGEA12 | RUNX3 | HLA-DQA1 |
| CCR5 | CXCL10 | IDO1 | MAGEA4 | S100A12 | HLA-DRB1 |
| CD14 | CXCL11 | IFI27 | MAGEC2 | S100A8 | HLA-E |
| CD163 | CXCL12 | IFI35 | MELK | S100A9 | OAZ1 |
| CD19 | CXCL13 | IFI6 | MKI67 | SDHA | PF4 |
| CD1C | CXCL5 | IFIH1 | MLANA | SELL | PRR5 |
| CD2 | CXCL8 | IFIT1 | MLH1 | SH2D1A | STK11IP |
| CD209 | CXCL9 | IFIT2 | MMP9 | SIGLEC5 | TBC1D10B |
| CD244 | CXCR2 | IFIT3 | MRC1 | SLAMF7 | TPSAB1 |
| CD247 | CXCR3 | IFITM1 | MS4A1 | SNAI1 | UBB |
| CD27 | CXCR4 | IFITM2 | MS4A2 | SPIB |  |
| CD274 | CXCR6 | IFNG | MS4A4A | STAT1 |  |
| CD276 | CYBB | IL10 | MSH2 | STAT3 |  |
| CD28 | DLL4 | IL10RA | MSH6 | STAT4 |  |
| CD38 | EGFR | IL12RB2 | MTOR | TAP1 |  |
| CD3D | EIF2AK2 | IL15 | MX1 | TBP |  |
| CD3E | ENTPD1 | IL17A | MYC | TBX21 |  |
| CD3G | EOMES | IL18 | NBN | TCL1A |  |
| CD4 | FAS | IL1A | NCAM1 | TDO2 |  |
| CD40 | FASLG | IL1B | NCR1 | TFRC |  |
| CD40LG | FCAR | IL2 | NECTIN2 | TGFB1 |  |
