# Supplementary material for: Adrenomedullin Inhibits the Efficacy of Combined Immunotherapy and Targeted Therapy in Biliary Tract Cancer by Disrupting Endothelial Cell Functions
Source: J Cell Mol Med. 2025 Mar 12;29(5):e70460. doi: 10.1111/jcmm.70460 (PMC11903196; doi:10.1111/jcmm.70460)
Supplement: Supplementary file 2 — Data S2 [file JCMM-29-e70460-s002.docx]

**Adrenomedullin inhibits the efficacy of combined immunotherapy and targeted therapy in biliary tract cancer by disrupting endothelial cell functions**

**Supplementary Methods**

1. **Quantitative real time PCR (qRT-PCR)**

| stage1 | premutability | Reps:1 | 95℃ | 30sec |
| --- | --- | --- | --- | --- |
| stage2 | cyclic response | Reps:40 | 95℃  60℃ | 10sec  30sec |
| stage3 | melting curve | Reps:1 | 95℃  60℃  95℃ | 15sec  60sec  15sec |

1. **Quality Control Measures for RNA Integrity**
   (a) Prevention of RNA Degradation During Extraction: RNA extraction from tissues should be performed using buffers containing RNAase inhibitors (DEPC-treated water) to prevent RNA degradation by RNAase.
   (b) RNA Concentration and Purity Assessment: The concentration of extracted RNA is measured using a spectrophotometer (NanoDrop), with the A260/A280 ratio typically ranging from 1.8 to 2.0.
   (c) Prevention of RNA Degradation: Post-extraction, RNA samples should be immediately stored at -80°C or rapidly frozen with liquid nitrogen to prevent degradation.
2. **Quality Control Measures for Protein Integrity**
   (a) Prevention of Degradation During Protein Extraction: Proteins were extracted using buffers containing protease inhibitors (PMSF) to prevent degradation.
   (b) Protein Concentration and Purity Assessment: Protein concentration was determined using the Bicinchoninic Acid (BCA) assay.
   (c) Prevention of Denaturation During Protein Storage: Extracted proteins were aliquoted and stored at -80°C to prevent denaturation and aggregation.
3. **Storage and reconstitution of ADM peptide**

Lyophilized recombinant proteins require reconstitution prior to aliquoting, storage, or experimental use. The lyophilized powder should first be centrifuged at 10,000-12,000 rpm for 30 seconds to collect any protein adhering to the tube walls or cap. Following centrifugation, the provided reconstitution buffer should be added to the lyophilized powder. Gentle mixing should be performed using a pipette or by inverting the capped tube several times to ensure proper resuspension. If necessary, brief low-speed centrifugation may be conducted. The protein should be resuspended to a minimum concentration of 100 μg/mL, with vigorous vortexing strictly avoided. The reconstituted protein solution should be maintained at room temperature for several minutes to ensure complete dissolution. For short-term applications (≤7 days), the reconstituted protein solution prepared with reconstitution buffer can be stored at 2-8°C. Direct freezing of freshly reconstituted protein should be avoided. For long-term storage, the protein should be reconstituted in a stabilizing solution containing either 0.1% BSA, 5% HSA, 10% FBS, or 5% trehalose. In serum-free experimental conditions, 5% trehalose should be used as the stabilizing agent. The reconstituted protein should be aliquoted and stored at -20°C to -80°C, where it remains stable for 3-6 months.

1. **Multiple fluorescence immunohistochemistry (mIHC)**

Paraffin-embedded tissue samples were sectioned into 3-5 μm thick slices using a microtome and mounted on slides. Target protein labeling was performed using the TSA fluorescence kit (Melady® Biosciences) following the manufacturer's instructions. The sections were baked at 60°C for 1 hour, deparaffinized in xylene, and rehydrated through a graded ethanol series (100%, 95%, 80%, and 70%) before being rinsed in ddH2O. Antigen retrieval was achieved by microwaving the sections in pH 9.0 EDTA buffer. Endogenous peroxidase activity was quenched with 3% hydrogen peroxide, and the slides were blocked with a blocking solution for 20 minutes and air-dried. The sections were incubated overnight at 4°C with the primary antibody. After incubating with a species-specific HRP-conjugated secondary antibody at room temperature for 20 minutes, the slides were washed with PBS and incubated with the appropriate fluorescent dye for TSA staining. The primary antibodies used were: CD4 (1:500, ab183685, Abcam), CD8 (1:500, ab217244, Abcam), CD31 (1:5000, ab182981, Abcam), HIF-a (1:200, 20960-1-AP, Proteintech), ADM (1:50, 10778-1-AP, Proteintech), and VE-cadherin (1:200, 66804-1-Ig, Proteintech). After TSA staining for the first antibody, the process was repeated for the second antibody. Following staining of all targets, the slides were washed with PBS and counterstained with DAPI. Finally, the slides were mounted with an appropriate mounting medium and scanned using a multi-channel fluorescence scanner (3DHISTECH, Pannoramic MIDI), with images analyzed using SlideViewer software.

1. **Analysis of multiplex immuno-stained slides**

Images were imported and rigorously examined using the AP-TIME pathological image analysis software (3D Medicines Inc.). Cell density was quantified by the software and expressed as the number of positively stained cells per square millimeter (cells/mm²). Furthermore, the software performed quantitative analysis of marker signals across the entire tumor tissue section and within specific regions of interest, yielding the number of positive cells and the expression levels of corresponding markers per cell in the analyzed regions. Fluorescence was considered across single or multiple channels as appropriate. Precise cell segmentation was achieved by establishing cell positivity thresholds for specific markers. To enhance visualization and interpretation, composite images representing the regions of interest were generated by the software based on the collected data. Subsequent data analyses were conducted using GraphPad Prism 9.0 software. Statistical significance between the two groups was determined using t-tests, with a p-value of less than 0.05 considered statistically significant.

1. **Drug dose-response assay**

HUVECs were plated in 96-well plates at a density of 1,000 cells per well and incubated overnight at 37°C with 5% CO₂ to allow cell attachment. Cells were then treated with Apatinib (13342S; MedChemExpress), ADM (P74426; Med Chem Express), ADM22-52 (P1471; MedChemExpress), or DMSO control (Y0320; MedChemExpress) at varying concentrations, with each concentration tested in triplicate. After 48 hours of treatment, 10 μL of CCK-8 reagent (Beyotime Biotechnology) was added to each well, followed by a 2-hour incubation at 37°C. Absorbance was measured at 450 nm using a microplate reader (Multiskan Sky, Thermo Fisher), and cell viability was normalized to the untreated control group. Dose-response data were fitted to a four-parameter logistic model using GraphPad Prism (version 9.0) to determine the half-maximal inhibitory concentration (IC₅₀). All experiments were performed in triplicate, and results are expressed as the mean ± standard deviation (SD) of three independent experiments.
